# Optimization of an *in situ* liver perfusion method to evaluate hepatic function of juvenile American alligators (*Alligator mississippiensis*)

**DOI:** 10.1242/bio.060532

**Published:** 2024-08-27

**Authors:** Yu Umeki, David Hala, Lene Hebsgaard Petersen

**Affiliations:** Department of Marine Biology, Texas A&M University at Galveston, 200 Seawolf Parkway, Galveston, TX, 77553, USA

**Keywords:** Crocodilian, Hepatic physiology, Hypoxia, Lactate, Pyruvate, Aspartate transferase

## Abstract

American alligators (*Alligator mississippiensis*) are a sentinel species whose health is representative of environmental quality. However, their susceptibility to various natural or anthropogenic stressors is yet to be comprehensively studied. Understanding hepatic function in such assessments is essential as the liver is the central organ in the metabolic physiology of an organism, and therefore influences its adaptive capability. In this study, a novel liver perfusion system was developed to study the hepatic physiology of juvenile alligators. First, a cannulation procedure was developed for an *in situ* liver perfusion preparation. Second, an optimal flow rate of 0.5 ml/min/g liver was determined based on the oxygen content in the effluent perfusate. Third, the efficacy of the liver preparation was tested by perfusing the liver with normoxic or hypoxic Tyrode's buffer while various biomarkers of hepatic function were monitored in the effluent perfusate. Our results showed that in the normoxic perfusion, the aspartate transferase (AST) and lactate/pyruvate ratio in the perfusate remained stable and within an acceptable physiological range for 6 h. In contrast, hypoxia exposure significantly increased the lactate/pyruvate ratio in the perfusate after 2 h, indicating an induction of anaerobic metabolism. These results suggest that the perfused liver remained viable during the perfusion period and exhibited the expected physiological response under hypoxia exposure. The liver perfusion system developed in this study provides an experimental framework with which to study the basic hepatic physiology of alligators and elucidate the effects of environmental or anthropogenic stressors on the metabolic physiology of this sentinel species.

## Teaser

**Summary:** A novel liver perfusion system was developed to study the hepatic metabolic capacity of American alligators.

## INTRODUCTION

Organ perfusion systems are commonly used to assess the effects of natural (e.g. temperature, hypoxia, pH) or anthropogenic (e.g. chemical pollutants) stressors on tissue-specific injury and associated physiological responses (Bessems et al., 2006; Brunengraber et al., 1973; Ghazi-Khansari et al., 2005; Lemasters et al., 1981; Nichols et al., 2009, 2013; Petersen and Gamperl, 2010; Von Bahr et al., 1970). One of the first organ perfusion systems established was the isolated mammalian heart perfusion system by Oscar Langendorff in 1895, to understand cardiac physiology (Bell et al., 2011; Skrzypiec-Spring et al., 2007; Zimmer, 1998). Since then, heart perfusion systems have provided several important breakthroughs in cardiac physiology (Bell et al., 2011; Skrzypiec-Spring et al., 2007; Zimmer, 1998). Such achievements were possible due to the tractable nature of the organ perfusion system, namely, its ability to sustain the physiological functions of the isolated organ, and its detachment from the rest of the body structure (e.g. no nervous innervation) enabling easy manipulation of the perfusate composition to study organ functions under typical versus atypical (or stressed) conditions (Bell et al., 2011; Skrzypiec-Spring et al., 2007; Zimmer, 1998).

While the heart is probably the most extensively studied organ using perfusion systems, another organ that has been the focus of study is the liver. The liver is a multifunctional organ that is intricately interrelated with other organs and tissues in the organism. The liver is known as the largest and expandable visceral organ that is heavily vascularized with both arterial and venous vessels (i.e. hepatic artery and portal vein) (Eipel, 2010; Lautt and Greenway, 1987). The liver is also the most metabolically active organ, responsible for performing a myriad of chemical reactions that metabolize or synthesize metabolic substances. For instance, hepatocytes primarily catalyze glycogen synthesis, gluconeogenesis, deamination, protein synthesis, and β-oxidation (Cameron and Sakhaee, 2007; Camiener and Smith, 1965; Charlton, 1996; Hatting et al., 2018; Houten and Wanders, 2010; Mitra and Metcalf, 2012; Nawaz et al., 2021; Nguyen et al., 2008; Sullivan et al., 2014). Additionally, the metabolism and elimination of drugs or foreign chemicals (xenobiotics), hormones, and other foreign substances are primarily mediated by biotransformation enzymes that are highly enriched in hepatocytes (Mitra and Metcalf, 2012). These compounds are metabolized to more hydrophilic metabolites and excreted into urine or bile (Mitra and Metcalf, 2012).

Given the significance of the liver for an organism's metabolic physiology, isolated liver perfusion systems have been developed to characterize and elucidate the functions of this vital organ under diseased or stressed conditions. For example, the hepatic perfusion system has contributed to our understanding of the basic physiological properties of the liver, such as phagocytosis of Kupffer cells, metabolism of energy sources (e.g. glucose and fatty acids), bile secretion, and the biotransformation and elimination of xenobiotics in animal models (e.g. rats, fish, etc.) (Brauer and Pessotti, 1952; Brunengraber et al., 1973; Goswami and Saha, 1998; Inoue et al., 2001; Rüdell et al., 1987; Sambri et al., 1996). This experimental approach has greatly advanced our comprehension of hepatic injury accompanied by the ischemic or hypoxic state that can be caused by cardiovascular disease or toxicant exposure (Araki and Lefer, 1980; Lemasters et al., 1981, 1983).

In terms of intertaxonomic application, liver perfusion studies have mostly been conducted using rats and pigs due to their accessibility, anatomical simplicity, or similarity to human physiology (Brauer and Pessotti, 1952; Brunengraber et al., 1973; Eiseman, 1961; Foth et al., 1991; Imber et al., 2002; Inoue et al., 2001; Nassar et al., 2014; Pang and Terrell, 1980; Rane et al., 1977; Reed et al., 1982a; Rüdell et al., 1987; Sambri et al., 1996; Xu et al., 2012). At present, the use of this system to study other taxa is generally limited with a few exceptions such as cats and select fish species (Araki and Lefer, 1980; Forlin and Andersson, 1981; Goswami and Saha, 1998; Nichols et al., 2009, 2013; Reed et al., 1982a).

American alligators [*Alligator mississippiensis* (Daudin, 1802)] are semi-aquatic reptiles that inhabit the fresh or brackish water along the Southeast coast of the USA (Fujisaki et al., 2014). Taxonomically, the family Alligatoridae along with Crocodylidae and Gavialidae belongs to the order Crocodilia, and American alligators (hereafter referred to as “alligators”) are one of several species in the family Alligatoridae (Green et al., 2014). They are often referred to as a sentinel species in the ecosystem due to their high trophic level and longevity (i.e. 30-75 year life span) (Milnes and Guillette, 2008; Weigl, 2014; Wilkinson et al., 2016). A sentinel species reflects the health of its ecosystem as it is exposed to the same stressors (artificial or natural) that may afflict the environment. Alligators have been used as an animal model for studying environmental quality as it has been shown to be susceptible to environmental stressors such as salinity change (Faulkner et al., 2018, 2019, 2021) and legacy pollutants (i.e. persistent organic pollutants, such as organochlorine pesticides) exposure (Crain and Guillette, 1998; Guillette et al., 1994; Milnes and Guillette, 2008).

To the best of our knowledge, a liver perfusion system has not previously been used to study crocodilian hepatic physiology, although a heart perfusion system has been developed to characterize the unique cardiac physiology of crocodilians (as diving reptilians) (Axelsson and Franklin, 1995; Franklin and Axelsson, 1994; Kohmoto et al., 1997). To that end, the goal of this study was to develop and optimize a liver perfusion system to study the hepatic metabolic capacity of juvenile alligators. We hypothesized that the *in situ* liver perfusion system of alligators would exhibit a shift from aerobic to anaerobic metabolism under hypoxic conditions that were used to demonstrate the validity and integrity of the organ perfusion system and hepatocytes. During perfusion, the tissue viability and metabolic activity of the liver were evaluated by measuring liver specific enzyme activity of aspartate transferase (AST) as well as organic metabolites such as lactate and pyruvate. AST was used as an indicator of hepatic injury (Amacher, 2002; Ozer et al., 2008), whereas an increase in lactate/pyruvate ratio under hypoxia was assumed to represent the onset of anaerobic metabolism (Alberti, 1977; Arteel et al., 1998).

## RESULTS

### Optimization of perfusion flow rate

The oxygen level in the influent perfusate was consistent at 1.00±0.01 mM (mean±standard error of the mean, s.e.m.; equivalent to 32.13 mg O_2_/L) throughout the experimental period across all flow rates. In the effluent perfusate, the oxygen content increased proportionally to the flow rate and reached a stable state faster with greater flow rates (Fig. 5). The mean±s.e.m. oxygen content at 0.25, 0.5, and 1.0 ml/min/g liver was 0.11±0.01, 0.19±0.00, and 0.22±0.00 mM (equivalent to 3.43, 5.93, and 6.96 mg O_2_/L), respectively. The flow rate of 0.25 ml/min/g liver reached a stable state at 3.5 h of perfusion while a flow rate of 0.5 ml/min/g liver reached a steady state at 1.5 h and it took 1 h at a flow rate of 1.0 ml/min/g liver. The effluent oxygen levels at flow rates of 0.5 and 1.0 ml/min/g liver were approximately 2-fold greater than that of the lowest flow rate. After 6 h of perfusion, the liver preparation integrity at a flow rate of 1.0 ml/min/g liver showed visible degradation due to the expansion of the liver and edema in the surrounding connective tissues while the *in situ* preparation maintained visible integrity (remained not swollen without discoloration) with the other two flow rates.

### Hepatic enzyme activity and glycolytic metabolites in baseline buffer perfusion

The AST activity was stable at 23.8±2.7 U/L (mean±s.e.m.) throughout 6 h of the baseline buffer perfusion period (Fig. 6A). There were no significant differences in lactate or pyruvate levels or lactate/pyruvate ratios during the experimental period (Fig. 6B, C). The mean±s.e.m. across all sampling points for lactate and pyruvate were 0.97±0.18 mM and 0.04±0.01 mM, respectively. The lactate/pyruvate ratio averaged 29.8±13.7 (mean±s.e.m.) across 6 h of baseline buffer perfusion.

### Hepatic enzyme activity and glycolytic metabolites in hypoxic buffer perfusion

The two-way ANOVA suggested significant differences between normoxia and hypoxia, however, the post-hoc *t*-test with Benjamini-Hochberg adjustment suggested no significance at each sampling point between the two treatments. The mean±s.e.m. AST activities in normoxia and hypoxia treatments were 17.3±5.7 and 22.7±4.7 U/L, respectively (Fig. 7).

The lactate level significantly increased (*P*<0.05) after 1 and 2 h of hypoxia exposure (1.71±0.26 and 2.32±0.44 mM) from the base level at 1 h of normoxia (0.66±0.03 mM) and 0 h of hypoxia (1.14±0.24 mM) (Fig. 8A). The pyruvate concentration showed no change between the two treatments (0.06±0.01 mM; mean±s.e.m. of all time points for normoxic and hypoxic perfusions) (Fig. 8A). The lactate/pyruvate ratio was 39.4±14.8 at 2 h of hypoxia which was significantly higher (*P*<0.05) than after 1 h (13.2±1.8) of normoxia or 0 h of hypoxia (13.0±2.4) (Fig. 8B).

### Biochemistry parameters analysis in normoxic and hypoxic perfusates

The glutamate dehydrogenase (GLDH) level was below the detection limit (<2 U/L) at all four time points in both normoxic and hypoxic buffer perfusions. Furthermore, the glucose level remained consistent between the four time points at 14.3±0.45 mM in both normoxic and hypoxic buffer perfusions.

## DISCUSSION

### Optimization of the cannulation procedure in the *in situ* alligator liver preparation

To the best of our knowledge, the present study describes the first ever development and application of a crocodilian *in situ* liver perfusion system to study the hepatic physiology of juvenile alligators. The alligator liver largely differs from mammals in gross anatomy and vasculature (Grigg and Kirshner, 2015; Reese, 1914; Van der Merwe and Kotze, 1993; Wyneken, 2011). These differences, especially in the vasculature system, establish the alligator liver perfusion system as a more complex system than that found in mammals. In a rat liver perfusion preparation, the hepatic portal vein and the postcava are usually cannulated for influent and effluent flow, respectively (Bessems et al., 2006; Inoue et al., 2001). These vessels are relatively easy to access, and the preparation can be isolated from the body cavity without any perfusate leakage if the free end of the postcava is ligated. In the alligator liver perfusion preparation, however, three veins (i.e. right and left abdominal veins, and postcava) require cannulation and the postcava is only approachable via the right atrium due to its anatomical position. Furthermore, the alligator preparation needs to be ligated at the caudal side of the liver to prevent unwanted perfusate flow to the peritoneal organs. The liver cannot be removed from the body cavity as an isolated organ system as it can damage the venous and arterial branches between them, resulting in flow leakage. The complex cannulation procedures highlight the importance of this study in establishing methods needed to develop an *in situ* liver perfusion system in alligators as other animal models (e.g. rat or fish) are not comparable to alligator hepatic physiology.

### Optimization of perfusion flow rate

In the optimization process of the liver perfusion system, the perfusion flow rate is one of the most important factors to control to ensure proper perfusion of the organ. A higher perfusion rate can cause tissue damage to sinusoidal endothelial cells (and compromise the sinusoidal fenestrations) due to high pressure, while a lower perfusion rate can lead to heterogenous distribution of buffer resulting in partial perfusion of the organ (Bessems et al., 2006). Therefore, setting an optimal flow rate is crucial to simulate physiological conditions for the liver and to accurately evaluate the organ functions.

The present study tested three flow rates: 0.25; 0.5; and 1.0 ml/min/g liver. The flow optimization trials showed that the three oxygen concentrations in the effluent reached 0.2 mM at the first time point (30 min) and stayed stable after 1 or 1.5 h at flow rates of 0.5 and 1.0 ml/min/g liver. However, after the entire perfusion period of 6 h, the flow rate of 1.0 ml/min/g liver resulted in less preserved liver integrity as determined by the visible expansion of the liver and severe edema in the connective tissues surrounding the liver.

The flow rates tested were selected based on estimated hepatic flow in alligators and recommended flow rates from other studies. For example, the hepatic blood flow in alligators was estimated based on the blood distribution in Burmese pythons (*Python molurus*) (Secor and White, 2010), as information on crocodilians was not available. Secor and White (2010) reported that the hepatic portal vein flow in Burmese pythons accounted for 12.7% of the cardiac output in resting, fasted animals and 35.6% of cardiac output in animals at peak digestion after feeding (Secor and White, 2010). Assuming pythons and alligators share similar traits in the blood distribution dynamics in the liver as they are both opportunistic feeders, the hepatic blood flow of alligators can be estimated as approximately 6.9 ml/min – 19.1 ml/min in the size of animals used in this study. The cardiac output was adapted from a study by Joyce et al. (2018) with juvenile alligators having a cardiac output of 19.6 ml/min/kg at 30°C (Joyce et al., 2018). Previous studies (Crile et al., 1940; Else and Hulbert, 1981, 1985) and our preliminary studies determined liver weight to be approximately 2% of the body weight in juvenile alligators. Thus, the hepatic blood flow was estimated at a flow rate of 0.13–0.35 ml/min/g liver. However, the optimal perfusion flow rate may be greater than this range since flow rates higher than the physiological hepatic flow are normally recommended to compensate for the absence of oxygen carriers (e.g. red blood cells) in perfusate (Bessems et al., 2006). Additionally, some studies of ectotherm liver perfusion support approximating flow rates. Forlin and Andersson (1981) reported that a target oxygen concentration in the effluent perfusate was achievable for the rainbow trout liver at perfusion rates of 0.5–1.0 ml/min/g liver (Forlin and Andersson, 1981). In their study, the effluent target oxygen concentration was set at 0.2 mM for sufficient oxygen supply in the liver as suggested by previous studies with the perfused rat liver (Sies, 1978). It was also noted that the higher rates recommended for the rat liver perfusion (3–5 ml/min/g liver) are not suitable for the rainbow trout model as a target metabolite in the effluent was diluted below the detection limit (Forlin and Andersson, 1981). The flow rates for rats are also unlikely applicable to alligators given their significantly lower cardiac output and metabolic rate than those of rats (i.e. both are approximately 1:20 in ratio) (Coulson et al., 1977, 1989). In addition, maximum oxygen consumption by the skate liver occurred at 0.4 ml/min/g liver or greater (Reed et al., 1982a). Collectively, the three flow rates were selected from the range of estimated hepatic flow in alligators and recommended flow rates in previous ectotherm studies. The results of this study suggest that a flow rate of 0.5 ml/min/g liver satisfactorily oxygenates the organ with effluent oxygen of 0.2 mM while maintaining the preparation integrity. Based on these findings, it was determined to use an optimal flow rate (0.5 ml/min/g liver) for the perfused alligator liver.

### Baseline liver viability biomarkers

Once the cannulation procedure and the optimal flow rate were established, the viability of the liver perfusion system was evaluated by monitoring several biomarkers of hepatic viability in the effluent perfusate. In this study, aspartate transferase (AST) activity and lactate and pyruvate levels were used as biomarkers of optimal liver function.

AST is an enzyme that catalyzes the conversion of aspartate and α-ketoglutarate to glutamate and oxaloacetate, which plays an essential role in promoting glycolysis and electron transport in cytoplasm and mitochondria via the malate–aspartate shuttle (McGill, 2016). AST is present both in the cytoplasm and mitochondria and is often used as a hepatic injury biomarker due to its abundant distribution in the hepatocytes (McGill, 2016). Previous studies reported an increase in blood AST levels after liver injury in a clinical or research setting (Ghazi-Khansari et al., 2005; Kamiike et al., 1989; McGill, 2016; Zoppini et al., 2016). The enzyme is a liver-specific biomarker commonly used in reptile species, including American alligators (Bogan and Mitchell, 2015). The results of this study showed that the AST activity was stable over 6 h of the perfusion period (23.8±2.7 U/L). This activity range was lower than that in blood plasma reported by previous studies (136.0–240.0 U/L) by roughly an order of magnitude (Bogan and Mitchell, 2015; Faulkner et al., 2021). The lower range in perfusate is likely due to the physical and biochemical properties of the perfusate that are dissimilar to those of blood, such as the lower viscosity due to the absence of blood cells and plasma proteins that may cause different flow dynamics and interactions with the tissue. However, since the AST activity was detectable at similar levels throughout the perfusion and the purpose of the present study was to establish the liver perfusion system in alligators, the results observed were considered reasonable.

The release of the cytoplasmic AST typically correlates with low oxygen availability or ischemia followed by reperfusion, while the mitochondrial AST is released due to secondary cell necrosis (Kamiike et al., 1989). Kamiike et al. (1989) reported that in a perfused rat liver, anoxic perfusate increased the cytoplasmic AST leakage in the first 3 h of anoxia exposure whereas the mitochondrial AST appeared after 4 h of exposure. They also demonstrated that the cytoplasmic AST leakage acutely spiked over the first 2 h of reperfusion after ischemia while the mitochondrial AST slowly elevated in the first 18 h of reperfusion producing the secondary AST peak (Kamiike et al., 1989). In the present study, the time spent from the onset of the cannulation procedure (first incision in the skin) until the first sampling point was approximately 75 min (8–15 min until the first cannulation). The liver was perfused for the next 6 h with the normoxic buffer. The absence of AST elevation in the perfusate indicated that the liver did not experience significantly low oxygen content, ischemia/reperfusion, or secondary necrosis during the cannulation procedure or the subsequent perfusion period.

The analysis of glycolytic metabolites agreed with the result of AST activity. In aerobic metabolism, glucose is converted to pyruvate and subsequently introduced to the tricarboxylic acid (TCA) cycle as acetyl-CoA to be oxidized. The reduced equivalents generated in the TCA cycle generate ATP through the mitochondrial electron transport chain (while reducing O_2_ to H_2_O) (Hochachka and Somero, 2002). However, when oxygen supply is limited, oxidative metabolism switches to anaerobic metabolism, whereby pyruvate is converted to lactate (by the cytosolic lactate dehydrogenase enzyme), resulting in a greater lactate/pyruvate ratio (Alberti, 1977; Hochachka and Somero, 2002). This anaerobic metabolism is also observed in active reptilians performing a prolonged dive or exhaustive exercise (Baldwin et al., 1995; Grigg and Gans, 1993; Hartzler et al., 2006; Seymour et al., 1985; Weber and White, 1986). The *in vivo* lactate level has been reported in various reptile species (such as turtles and crocodilians) including alligators, and can range from 1.0–98 mM in animals at rest (Baldwin et al., 1995; Clark and Miller, 1973; Frankel et al., 1966; Gatten et al., 1991; Hartzler et al., 2006; Ornata Author and Gatten, 1975; Seymour et al., 1985). Specifically in alligators, 1.0 mM in whole animal homogenate, arterial blood, and plasma has been recorded (Coulson and Hernandez, 1979; Gatten et al., 1991; Hartzler et al., 2006). In contrast, little information exists on the pyruvate levels in reptiles. Frankel et al. (1966) and Clark and Miller (1973) reported the pyruvate levels in the brain, ventricular blood, and liver of red-eared sliders (*Trachemys scripta elegans*) under an oxygenated condition, averaging 21.3, 1.4, and 0.1 mM, respectively. While the lactate/pyruvate ratio in crocodilians is not known, the ratio in red-eared sliders can be estimated as 0.9–20 based on previous studies (Frankel et al., 1966; Clark and Miller, 1973).

In this study, the lactate concentration (0.97±0.18 mM) was comparable to the reported value in alligators, whereas the pyruvate concentration (0.04±0.01 mM) was roughly half of the reported value in the red-eared slider liver. While this value could be due to physiological variation in alligators, the difference with the turtle may be due to the pyruvate's lower stability in blood samples compared to lactate (Chuang et al., 2006; McCaughan et al., 2000; Seymour et al., 2011; Wulkan et al., 2001; Zavorsky et al., 2021). Pyruvate is known to decrease more rapidly in whole blood at 4°C (50% loss after 12 h) or in deproteinized blood at −20°C (more than 8% loss after 25 days) (Chuang et al., 2006; Wulkan et al., 2001) than lactate which remains stable at those storage temperatures (McCaughan et al., 2000; Seymour et al., 2011; Zavorsky et al., 2021). The instability of pyruvate is attributed to its chemical property as a 3-keto acid which spontaneously degrades to ketones, a dimer, or other polymetric derivatives (Yuzawa et al., 2018; Zhou, 2014). In the present study, the perfusate collected during the perfusion was temporarily stored on ice until it was centrifuged to pellet red blood cells (RBC) to avoid hemolysis, and the recovered supernatant was stored at −20°C at the end of the perfusion period. RBC relies on anaerobic metabolism and is enriched with lactate dehydrogenase (LDH), which facilitates mutual conversion of pyruvate and lactate (Chuang et al., 2006). Hemolysis in the blood sample results in a release of a large amount of LDH along with a false increase in the pyruvate concentration due to an elevated reaction rate between pyruvate and lactate (Chuang et al., 2006). Therefore, the removal of RBC was performed in this study although the RBC in perfusate was typically a very small amount. Given the length of the sample storage period on ice or in the freezer, which could be up to several hours or several months respectively, the observed lower concentration of pyruvate may be due to the compound's spontaneous degradation. For maximum preservation of pyruvate, it is recommended to store the sample (i.e. blood or equivalent) at room temperature and perform the assay in the next 12 h, or immediately deproteinize the sample and store it at 4°C or −20°C and perform the assay within 12 h or 15 days, respectively (Chuang et al., 2006; Wulkan et al., 2001). Such sample processing may need to be considered in further studies. Accordingly, it seems unlikely that the perfused liver was in an anaerobic metabolic state during the perfusion considering the measured lactate/pyruvate ratio (29.8±13.7) that was similar to the published value in the turtles and the potentially underestimated pyruvate levels in the perfusate.

### Evaluation of the impact of hypoxia on the perfused liver preparation

To evaluate the validity and robustness of the preparation, the perfused liver was exposed to a severe hypoxic condition after 2 h of normoxic buffer perfusion. The same parameters used in the baseline perfusion were measured to monitor liver injury (AST) and aerobic/anaerobic metabolism (lactate and pyruvate). There was no significant difference observed in the AST activity between normoxic and hypoxic periods. This result contradicts the observations from the perfused rat liver where the cytosolic AST activity soared (approximately 6×) after 2 h of exposure to anoxia (Kamiike et al., 1989). In our study, it is likely that the hypoxia exposure duration was not sufficiently long or severe enough to cause significant cell injury.

Exposure of the alligator livers to the hypoxic buffer resulted in significantly increased lactate levels in the perfusate (2.32±0.44 mM after 2 h of the treatment) compared to the baseline level after 1 h of normoxia perfusion (0.66±0.03 mM). The pyruvate concentrations in both treatments remained in the same range as that of the baseline study. Accordingly, the lactate/pyruvate ratio significantly increased at 2 h of hypoxia exposure, supporting our hypothesis. These results demonstrate the robustness of the liver and hepatic cells in that it shows the hepatic cells are able to respond to changes in perfusion conditions as would occur *in vivo*. It further shows that the cannulations perfused the entire liver and maintained the overall organ integrity.

Crocodilians are diving animals that are regularly exposed to hypoxia from a prolonged dive. Although most recorded voluntary dives in free ranging crocodilians are very short (i.e. less than 10 min in alligators) and conducted aerobically (Axelsson and Franklin, 2011; Grigg and Johansen, 1987; Wright, 1987), they can perform anaerobic activities when their aerobic metabolism capacity does not meet the energy needs. Previous studies showed that a “fright” dive (i.e. when a dive is forced, disturbed, or prolonged) or exhaustive exercise drastically increases lactate levels lowering pH in the animals (Bennett, 1982; Grigg and Gans, 1993; Smith et al., 1974; Wright, 1987). In alligators, the normal lactate level of 1.0 mM can quickly rise about 9-fold after 1 min of forced swimming (Gatten et al., 1991) and 16-fold with a decrease in pH from 7.4 to 7.0 after exhaustive activity (Hartzler et al., 2006) at 30°C. Therefore, the increase in lactate level in this study (2.6–3.5-fold) was in the physiological range suggesting a modest hypoxic effect.

In an anaerobic tissue, pyruvate is converted to lactate at a greater rate resulting in depletion of pyruvate and a high lactate/pyruvate ratio. For example, in anoxic freshwater turtle (*Pseudemys scripta elegans*) livers, lactate/pyruvate ratios of 100–500 have been recorded (Clark and Miller, 1973). In this study, the highest lactate/pyruvate ratio (39.4±14.8) was observed after 2 h of hypoxia exposure, which is approximately 3 times higher than the ratio observed in the normoxic perfusate. This increase in the ratio is not as drastic as the reported values in the anaerobic turtles. The relatively low lactate/pyruvate ratio is likely as the hypoxic effects may have been less intense, and no decline in the pyruvate level was measured in this study. It is possible that the pyruvate level did not decrease in this study as the perfusate contained a high concentration of glucose (15 mM), which is 3-fold higher than expected physiological levels (Faulkner et al., 2018, 2019; Hamilton et al., 2016; Kohmoto et al., 1997), and therefore the pyruvate pool was not depleted during the perfusion period. It is also possible that glucose supply from the liver storage increased as glycolysis was induced by the hypoxia exposure (Robey et al., 2005). Thus, to use the lactate/pyruvate ratio as a biomarker of hypoxia in the liver perfusion system, the hypoxic exposure needs to be intensified or prolonged, or the glucose level in influent may need to be lowered (or both) to ensure the pyruvate decline during anaerobic metabolism. Nonetheless, the viability of the preparation was demonstrated by the onset of anaerobic metabolism observed based on the increased lactate and lactate/pyruvate ratio.

### Perfusate biochemistry analysis

The effluent perfusate from an animal exposed to normoxia and hypoxia in the hypoxia perfusion study was analyzed for GLDH activity and glucose concentration. The results showed that the GLDH activity was below the detection limit (<2 U/L) whereas the glucose level remained consistent at 14.3±0.45 mM in all four samples (i.e. normoxia 0 and 120 min and hypoxia 0 and 120 min). GLDH has been reported as a liver specific enzyme in reptiles and is suggested to be a useful liver injury biomarker (Adamovicz et al., 2019; Eatwell et al., 2014; Leineweber et al., 2019). In this study, the GLDH activity was not detectable at all sampling points indicating no hepatic damage during the perfusion. This result is in alignment with the results of the AST activity discussed above. The glucose level was measured as an indicator of glycolytic metabolism and the detected concentrations were constantly similar to that in influent perfusate (15 mM). It is possible that the glucose consumption by the liver was not observable due to the large influx of glucose for alligators' lower metabolic rate than mammals (Coulson et al., 1989). Additionally, the liver may prefer fatty acids rather than glucose for energy production during hypoxia (Gracey et al., 2011; Zhang et al., 2017). For further work, a lower glucose level in the influent and targeted metabolite analysis may be of help to reveal liver specific energy metabolism in alligators (i.e. glycolytic metabolism capacity and preferred energy source under different conditions).

In summary, this study demonstrated that the cannulation and perfusion procedures were properly optimized to maintain the viability of the perfused liver to investigate hepatic functions in alligators. This organ perfusion system has diverse potential applications; such as the evaluation of impact of natural and anthropogenic stressors (e.g. temperature, salinity, and chemical pollutants), on the metabolic physiology of alligators. This system can aid in a better understanding of the hepatic physiology and pathology in alligators and other crocodilian species which are sentinels of environmental quality and ‘apex’ predators within the ecosystems they inhabit.

## MATERIALS AND METHODS

### Animal husbandry

Juvenile alligators (2–3 years old) [mean body mass±s.d.: 2700±356.3 g; snout-to-vent length (SVL): 45.9±2.0 cm] were kindly donated by the Rockefeller Wildlife Refuge (Grand Chenier, LA, USA). Three round fiberglass tanks (diameter: 177.8 cm; height: 29.5 cm) were used to keep 15 juvenile alligators in ∼600 L of fresh water, respectively. Each tank contained three slate platforms to allow animals to move in and out of the water, and each platform was fitted with an overhead reptile 160 W UV light heat lamp (Zoo Med Laboratories, Inc., San Luis Obispo, CA, USA) to maintain the ambient temperature at 22.7±0.5°C and support basking behaviors. The air temperature of the facility was kept at 21.7±0.2°C while the water temperature was at 20.6±0.3°C. Half of each tank was shaded with a tarp to allow animals refuge and a place to thermoregulate. Animals were fed with an average of 1% body weight of Mazuri^®^ Reptile Diet pellets (PMI Nutrition International, Shoreview, MN, USA) three times per week. Water changes were performed 24 h after each feeding by draining the water and waste of each tank and refilling it with carbon-filtered fresh water. All studies were approved by Texas A&M University IACUC under the AUP 2019-0095.

### Liver perfusion system

**Fig. 1.**
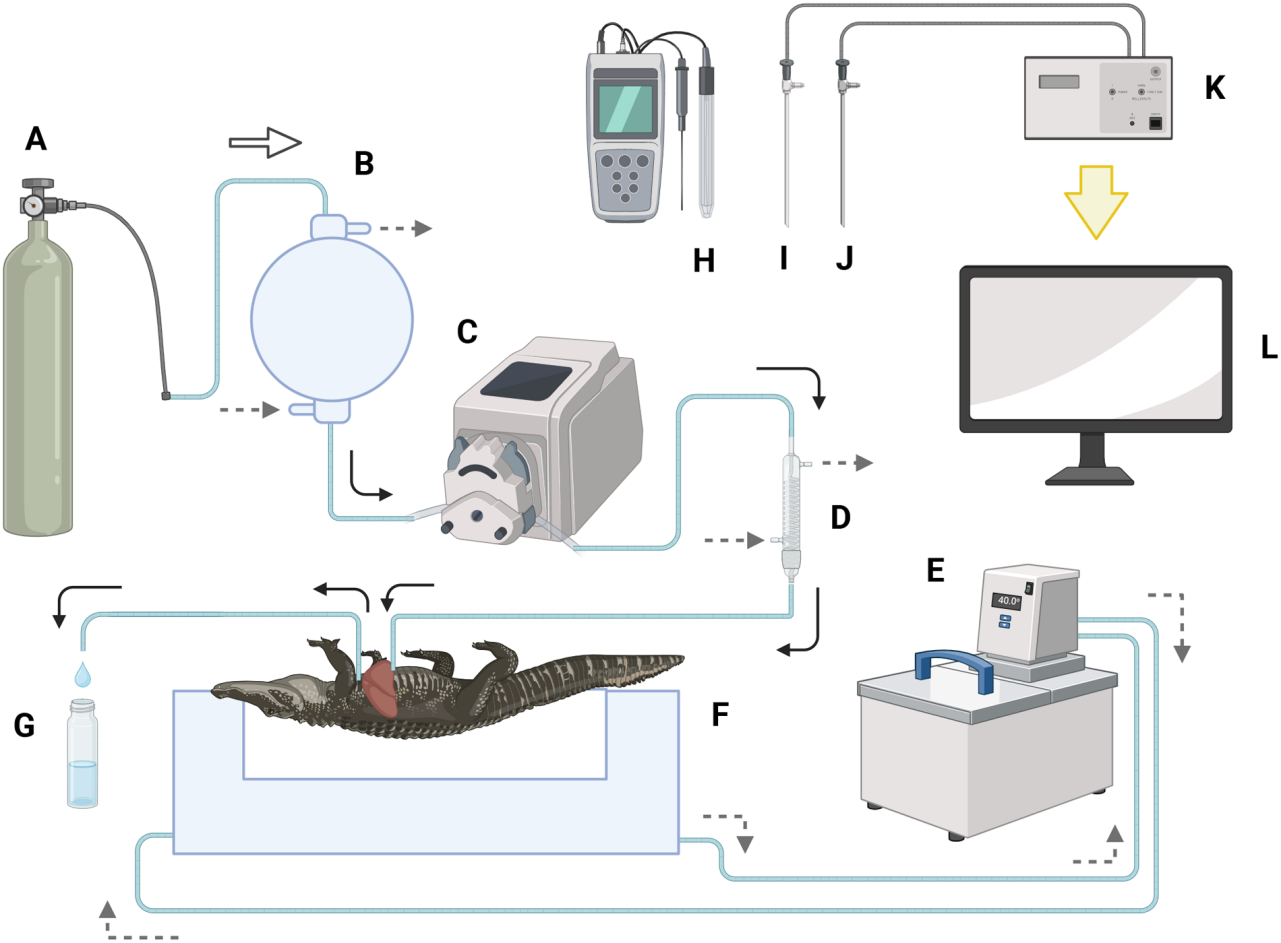
**Schematic diagram of alligator liver perfusion system.** (A) nitrogen or oxygen gas tank, (B) perfusate reservoir, (C) peristaltic pump, (D) bubble trap, (E) water bath circulator, (F) perfusion chamber, (G) sampling glass vial, (H) pH probe, (I) optical fiber oxygen probe, (J) temperature probe, (K) communicating component, and (L) computer screen. Solid arrow: perfusate flow, dotted arrow: water flow, open arrow: gas flow.

The perfusion apparatus was set up as a flow through system where the perfusion buffer flowed through the apparatus and the effluent was sampled for analysis at sampling points or otherwise discarded. The system consisted of a two-liter buffer reservoir (Southeastern Laboratory Apparatus, Inc., North Augusta, SC, USA), a peristaltic pump (Masterflex L/S: Cole Parmer, Vernon Hills, IL, USA), a bubble trap (Radnoti, Dunedin, New Zealand), a perfusion chamber (Northern Acrylics Inc., Duluth, MN, USA), and a water circulator bath (model 1196: PolyScience, Niles, IL, USA) (Fig. 1). The reservoir, bubble trap, and perfusion chamber were all water jacketed and connected with the water bath recirculation system to maintain the temperature at 28°C. A 95% O_2_: 5% CO_2_ gas mixture cylinder and a 100% N2 gas cylinder with a gas regulator were connected to airtight tubing (Tygon E-LFL and LFL: Saint-Gobain North America, Malvern, PA, USA), the end of which was attached to a stainless air stone (JoyTube, Amazon.com) that aerated the perfusion buffer in the reservoir. A second peristaltic pump (Mini Plus 3: Gilson, Middleton, WI, USA) was placed to continuously add 100 mM sodium hydroxide (at an average flow rate of approximately 4 ml/min) into the reservoir buffer to adjust to physiological pH. A pH probe (Oakton PC 700: Cole-Parmer North America, Vernon Hills, IL, USA), optic fiber dissolved oxygen probe (dipping probe mini sensor: Loligo Systems, Viborg, Denmark), temperature probe (Pt1000: Loligo Systems, Viborg, Denmark), and a handheld dissolved oxygen probe (550A: YSI, Yellow Springs, OH, USA) were placed in the reservoir during the experiment to continuously monitor buffer pH, oxygen levels, and temperature. Each of these parameters was also measured in the effluent buffer.

### Perfusion buffer for liver perfusion

Tyrode's buffer solution was selected as a perfusion buffer based on its previous use in an alligator heart perfusion preparation (Kohmoto et al., 1997). The buffer contained 144 mM NaCl (Sigma Aldrich, St. Louis, MO, USA), 5 mM KCl (Sigma Aldrich, St. Louis, MO, USA), 0.9 mM MgCl_2_·6H_2_O (Sigma Aldrich, St. Louis, MO, USA), 6 mM HEPES free acid (EMD Millipore, Burlington, MA, USA), 15 mM glucose (Sigma Aldrich, St. Louis, MO, USA), and 1.5 mM CaCl_2_.2H_2_O (Sigma Aldrich, St. Louis, MO, USA) in Milli-Q water (Kohmoto et al., 1997). The pH of the buffer was adjusted to 7.8 with 1 M NaOH (Sigma Aldrich, St. Louis, MO, USA) and stored at 4°C until use and warmed up to 28°C in a water bath on the day of the perfusion. The buffer was gassed with 95% O_2_ and 5% CO_2_ gas mixture (UN3156: Airgas, Radnor, PA, USA) for the baseline perfusion study, and normoxia (control) period of the hypoxic perfusion study while it was aerated with N_2_ gas (UN1066: Airgas, Radnor, PA, USA) to lower oxygen saturation during hypoxia period. The pH of the buffer was maintained between 7.4 and 7.6 by adding 100 mM or 1 M NaOH throughout the experiment as needed.

### Description of major organs and vasculature anatomy

**Fig. 2.**
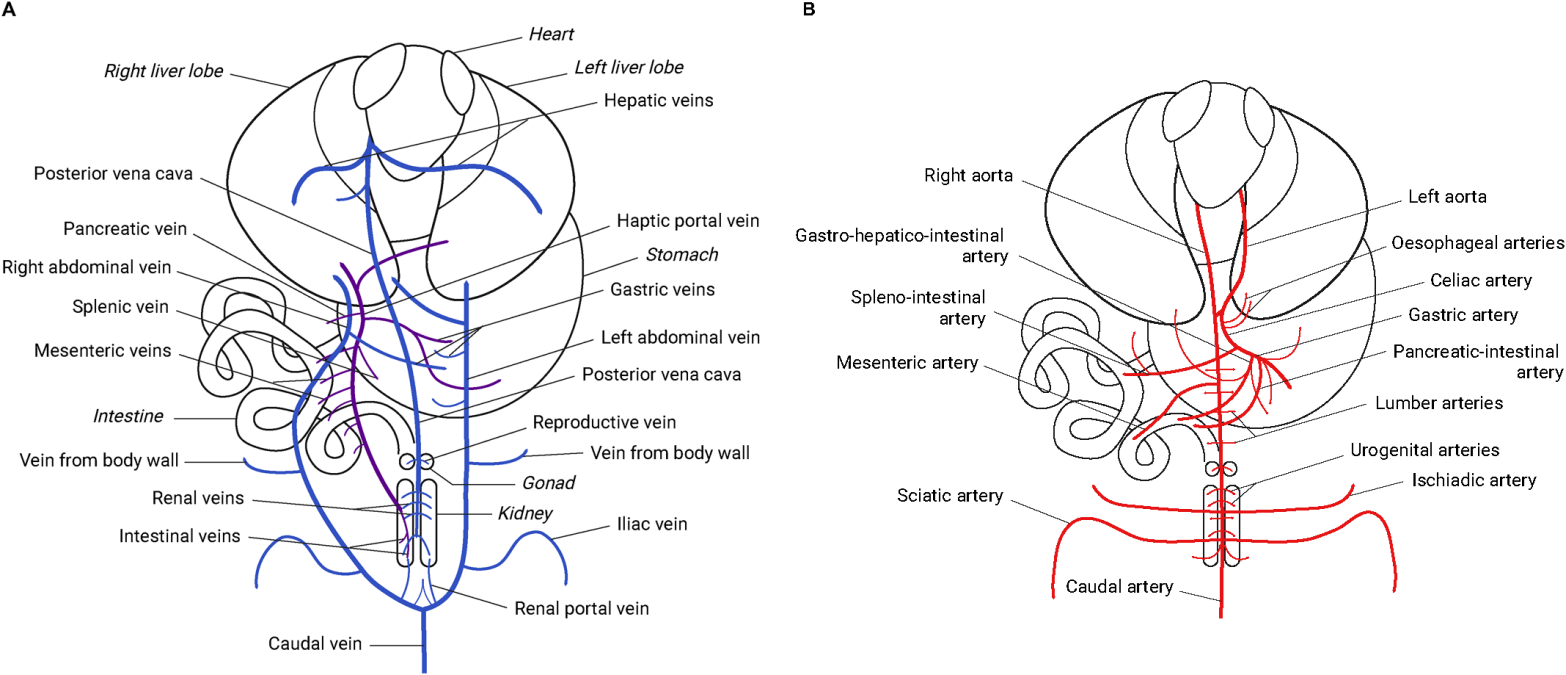
**Diagrams showing ventral view of (A) major organs and associated veins and (B) ventral view of major organs and associated arteries.** The liver receives venous blood via the right and left abdominal veins and hepatic portal vein, and arterial blood via the gastro–hepatico–intestinal artery. The blood is subsequently drained from the liver through the hepatic veins which empties into the sinus venosus via the posterior vena cava. Adapted from Grigg and Kirshner (2015) (after Reese, 1914; 1915). This image is not published under the terms of the CC-BY license of this article. For permission to reuse, please see Reese (1914; 1915).

In alligators, the liver is situated caudal to the heart and lungs (i.e. pleural cavity) and cranial to the digestive organs (i.e. peritoneal cavity) and contained within the coelomic cavity. The liver consists of two lobes that are connected by the isthmus and the right lobe is normally larger than the left. The hepatic vasculature in alligators consists of three veins (i.e. right and left abdominal veins and hepatic portal veins) and one artery (i.e. gastro–hepatico–intestinal artery) for blood supply and two hepatic veins for blood return (Fig. 2A,B). Both sides of the abdominal veins collect venous blood from other parts of the body before the liver, via the iliac veins, body wall veins, and gastric veins. The hepatic portal vein in alligators empties into the right liver lobe near the bile duct, receiving venous blood via branches from the pancreas, stomach, spleen, and mesentery attached to small and large intestines (Fig. 2A). The gastro–hepatico–intestinal artery arises from the celiac artery and carries oxygenated blood to the stomach, liver, and small intestine (Reese, 1914), although its hepatic branch was not distinct in the juvenile alligators studied (Fig. 2B). The flow from the liver is sent back to the heart via two hepatic veins. A large hepatic vein from each side of the liver lobe opens into the posterior vena cava close to the sinus venosus, which then empties into the right atrium (Fig. 2A). The posterior vena cava enters the dorsal posterior end of the right liver lobe, from which it receives several branches, after collecting blood from reproductive organs and kidneys. The sinus venosus is a thin-walled structure that is situated at the dorsal side of the right atrium. Venous blood enters the sinus venosus via three large vessels and one small vessel: the posterior vena cava (postcava), two anterior venae cavae (precavae), and the coronary vein, before the right atrium.

### Cannulation procedures of the *in situ* liver preparation

**Fig. 3.**
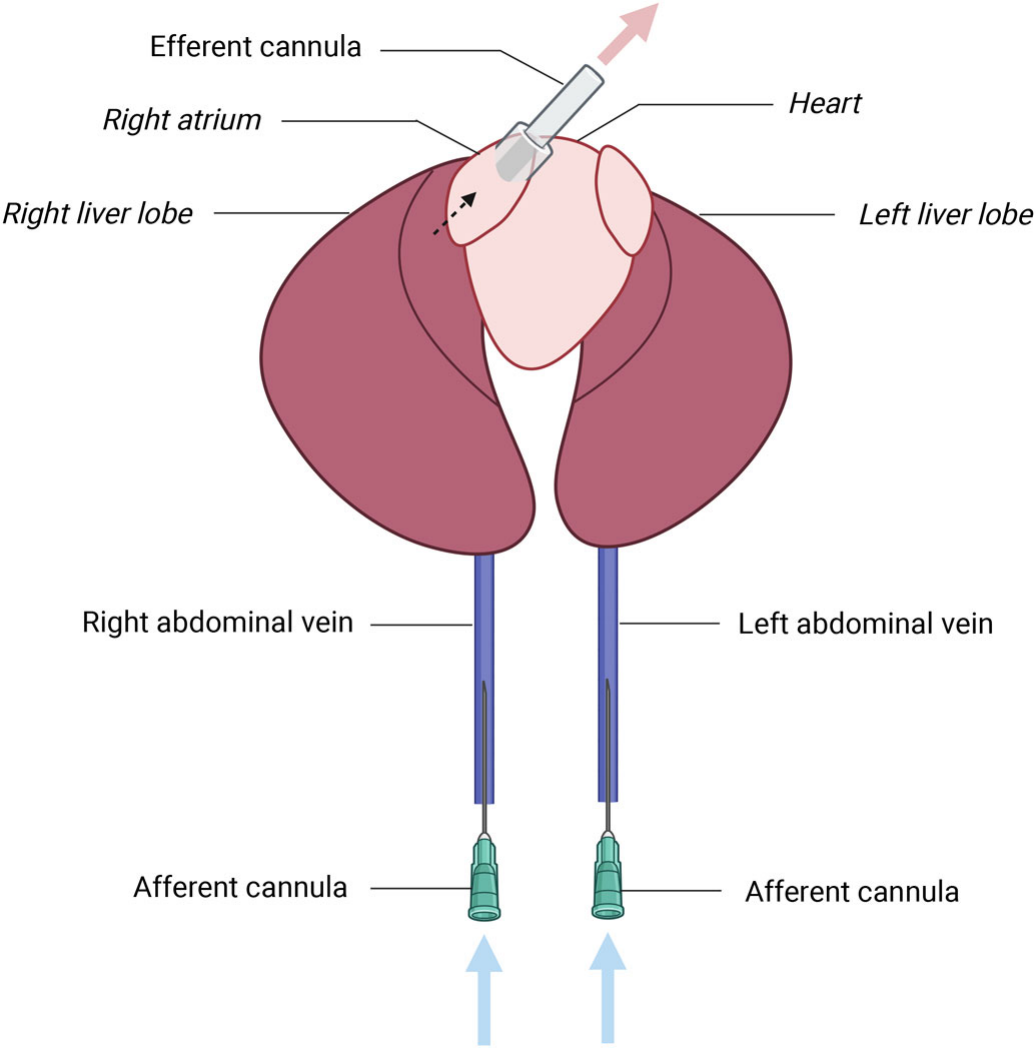
**Schematic image showing the location of major veins and anatomical structures of the liver.** The liver was perfused from the right and left abdominal veins through the hepatic veins which empties into the sinus venosus. The efferent cannula was inserted into the sinus venosus via the right atrium to collect the perfusate. Blue arrows: influent perfusate, red arrow: effluent perfusate, dotted arrow: perfusate flow from the posterior vena cava through sinus venosus.

A juvenile alligator was anesthetized using 2% isoflurane in an enclosed chamber. When unconsciousness was achieved and ventilation ceased, the animal was checked for eye blink and pedal reflexes. When the reflexes were absent, the alligator was cranially pithed with an 18-gauge needle. Cranial pithing was performed as an adjunctive measure of euthanasia and is commonly used in breath-holding diving animals. Once euthanized, an intravenous injection of heparin (100 U/kg) was given to the alligator via the intravertebral vein between thoracic vertebrae to prevent blood coagulation (Zippel et al., 2003). The alligator was then intubated to aerate the lungs at the rate of 60 breaths/min. The body cavity was opened and the left abdominal vein (LAV) was exposed, freed from connective tissue, and cannulated with a 21-gauge cannula (Corza Medical, formerly Surgical Specialties, Westwood, MA, USA) (Fig. 3). Tyrode's buffer was immediately infused through the cannula at half of full flow rate (i. e. 0.25, 0.5, or 1 mL/min/g liver for flow rate optimization and 0.5 mL/min/g liver for baseline and hypoxia studies). Subsequently, the right atrium (RA) was exposed and partially opened to flush out the blood from the liver and release the pressure of the buffer. The right abdominal vein was then exposed and cannulated as described for the LAV. The flow rate of the buffer was immediately increased to full flow as soon as cannulation of both abdominal veins was completed.

Next, the digestive system was ligated to isolate it from the liver. This approach was to retain the buffer flow into the liver and minimize any diversion into venous or arterial branches to the other organs. Finally, the posterior vena cava, which drains from the hepatic veins, was cannulated via the RA to collect the effluent perfusate. Once the cannulation procedure was completed, the *in situ* liver preparation was placed within a water-jacketed chamber to maintain the temperature at 28°C.

### Optimization of perfusion flow rate

To optimize the oxygen saturation of the liver tissue, different flow rates of influent perfusate were tested. Based on previous studies, 0.25 ml/min/liver gram, 0.5 ml/min/liver gram, and 1 ml/min/liver gram were selected (Brunengraber et al., 1975; Forlin and Andersson, 1981; Reed et al., 1982b). The flow rate was adjusted via the peristaltic pump that was connected to the buffer reservoir. The liver weight of the subject animal was calculated based on its body weight [the liver weight of reptiles normally accounts for 2–3% of the animal's body weight (Crile et al., 1940; Hernandez-Divers and Cooper, 2005; Kölle et al., 2022; Rogers and Dintzis, 2018)]. Liver mass was further determined as averaging 2% of body mass for juvenile alligators in this study during preliminary experiments (data not shown). Each flow rate was tested on one animal for 6 h respectively while physiological parameters including oxygen saturation in both influent and effluent perfusate were monitored. In addition, tissue integration of the perfusion preparation was visually observed during the experimental period (e.g. size and color of the organ).

### Baseline buffer perfusion

**Fig. 4.**
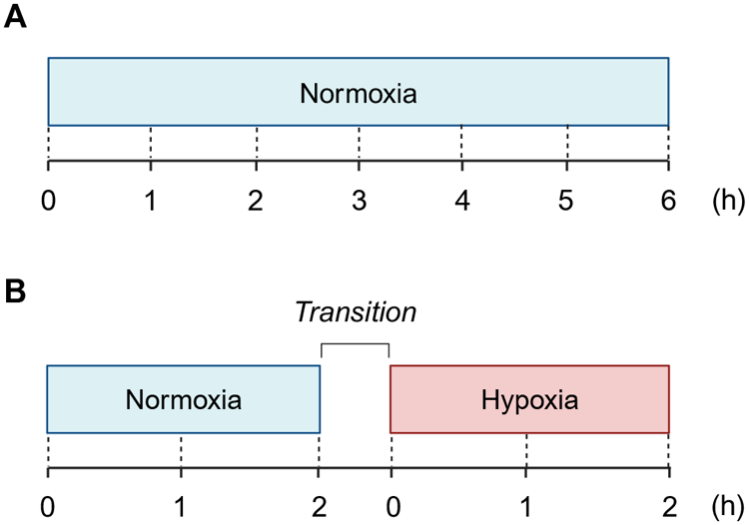
**Experimental protocols of (A) baseline study (*n*=4) and (B) hypoxia study (*n*=6).** Diagrams are showing the time course of each experiment. The baseline perfusion was run for 6 h, and the hypoxia perfusion was run for 4 h plus a transition period which comprised 10 min where oxygen levels were lowered. This was followed by 20 min of acclimation period to hypoxia.

The liver preparation was perfused under a ‘control’ condition for 6 h to establish baselines of physiological parameters and hepatic enzyme activity. pH, temperature, and dissolved oxygen in influent and effluent perfusate were recorded every 30 min for 6 h, to evaluate whether those values remained stable (Fig. 4A). The influent and effluent perfusates were stored on ice until centrifuged at 3000 rpm, 4°C for 10 min to pellet any residual red blood cells and the supernatant was stored at −20°C until further analyses. Upon completion of the 6 h perfusion, the sex of each animal was identified by examining the internal reproductive organs.

**Fig. 5.**
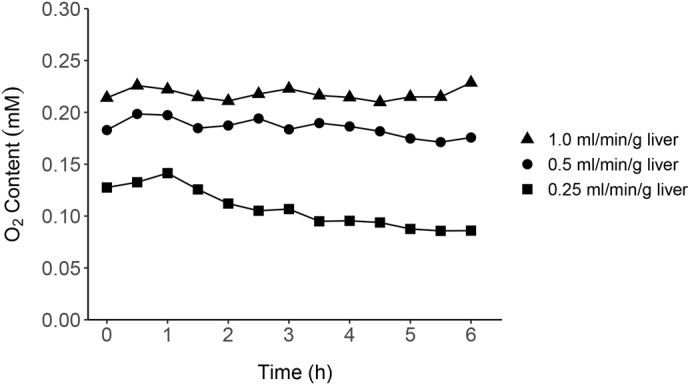
**Line graph showing the oxygen content in effluent perfusate at three different perfusion flow rates.** The flow rate of 0.25 ml/min/g liver reached the stable state at 3.5 h of perfusion whereas flow rates of 0.5 and 1 ml/min/g liver stabilized at 1.5 and 1 h, respectively. Each data point represents the measurement from an animal (*n*=1 for each flow rate).

**Fig. 6.**
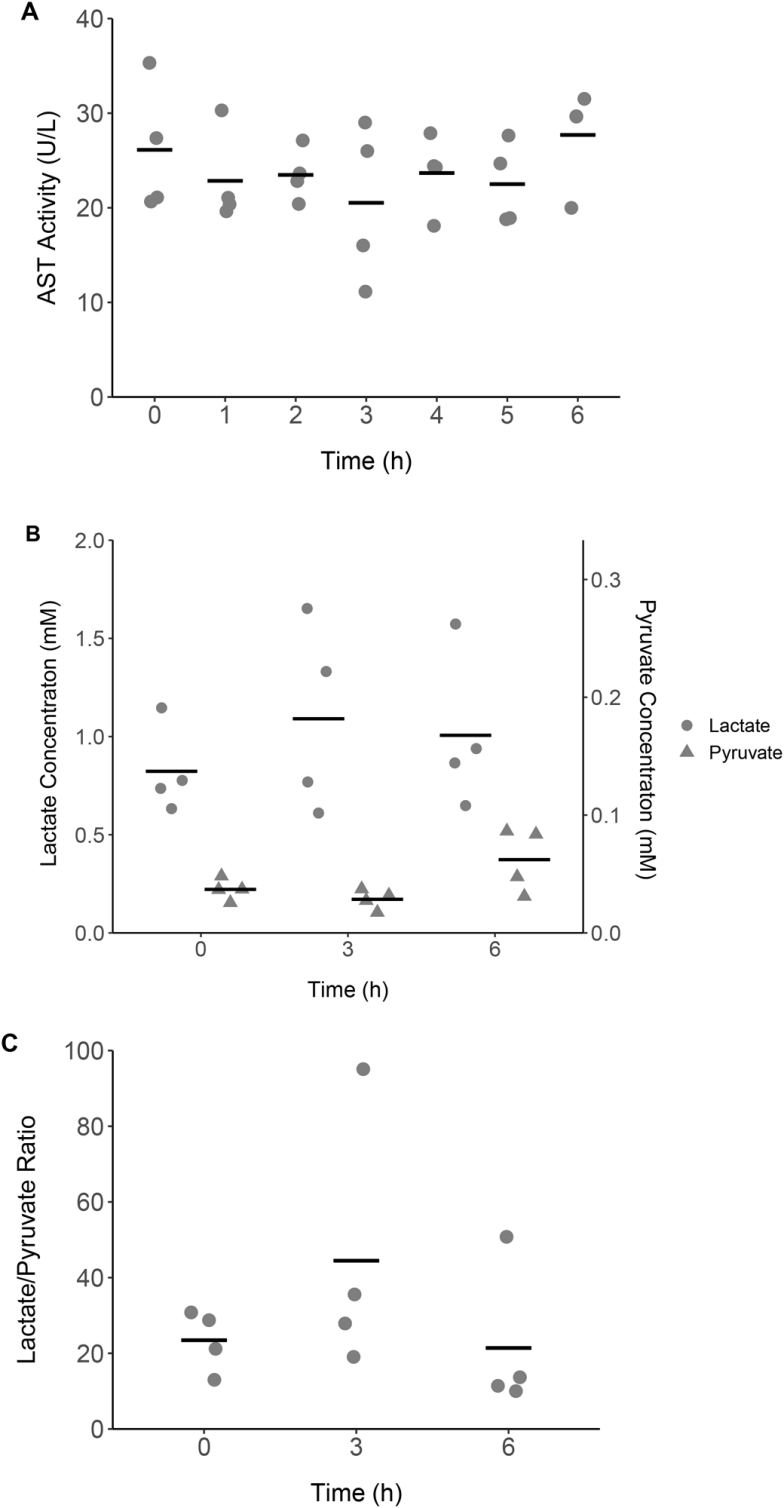
**Hepatic function biomarkers in baseline perfusate. (A) AST activity in effluent perfusate sampled hourly from the liver perfused with the baseline buffer for 6 h. (B) Lactate and pyruvate levels and (C) Lactate/pyruvate ratio in effluent perfusate after 0, 3, and 6 h of perfusion with the baseline buffer.** AST activity was stable during the 6 h perfusion period. There was no significant change in lactate, pyruvate, or lactate/pyruvate ratio during the perfusion with normoxic buffer (one-way ANOVA). Each data point represents the measurement from an animal and each bar shows a mean (*n*=4).

**Fig. 7.**
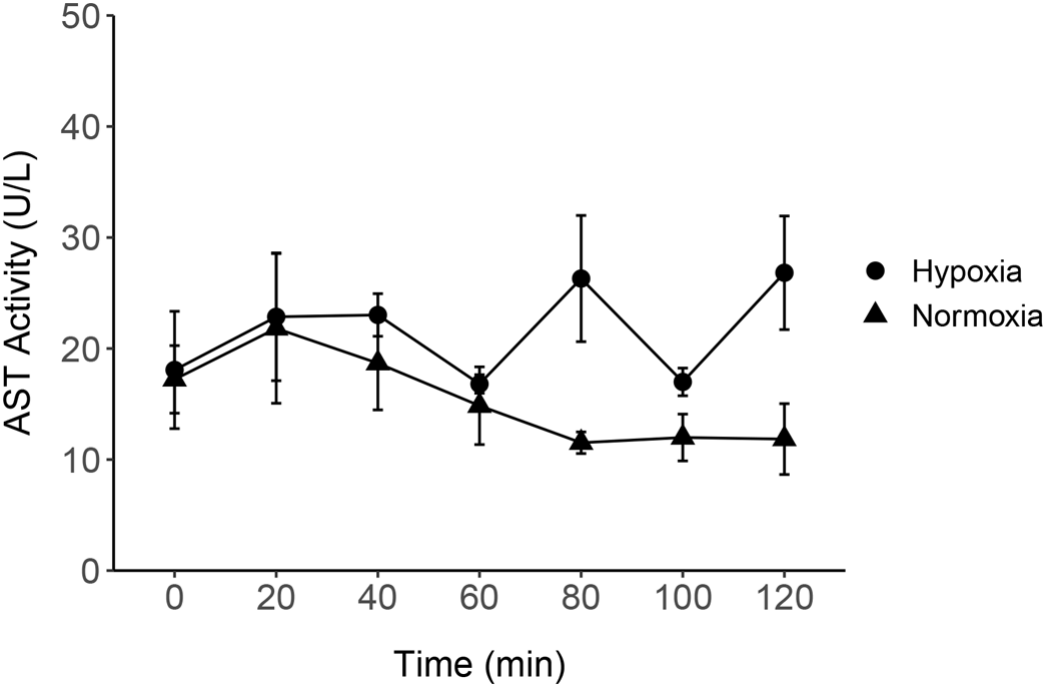
**Line graph showing AST activity in effluent perfusate sampled every 20 min for 2 h (120 min) of normoxic and hypoxic perfusion.** No significant change was observed in AST activity between normoxia and hypoxia samples (two-way ANOVA followed by *t*-test with BH adjustment). Data are mean±s.e.m. (*n*=6 except for *n*=5 at 0 min and 100 min and *n*=4 at 60 min).

**Fig. 8.**
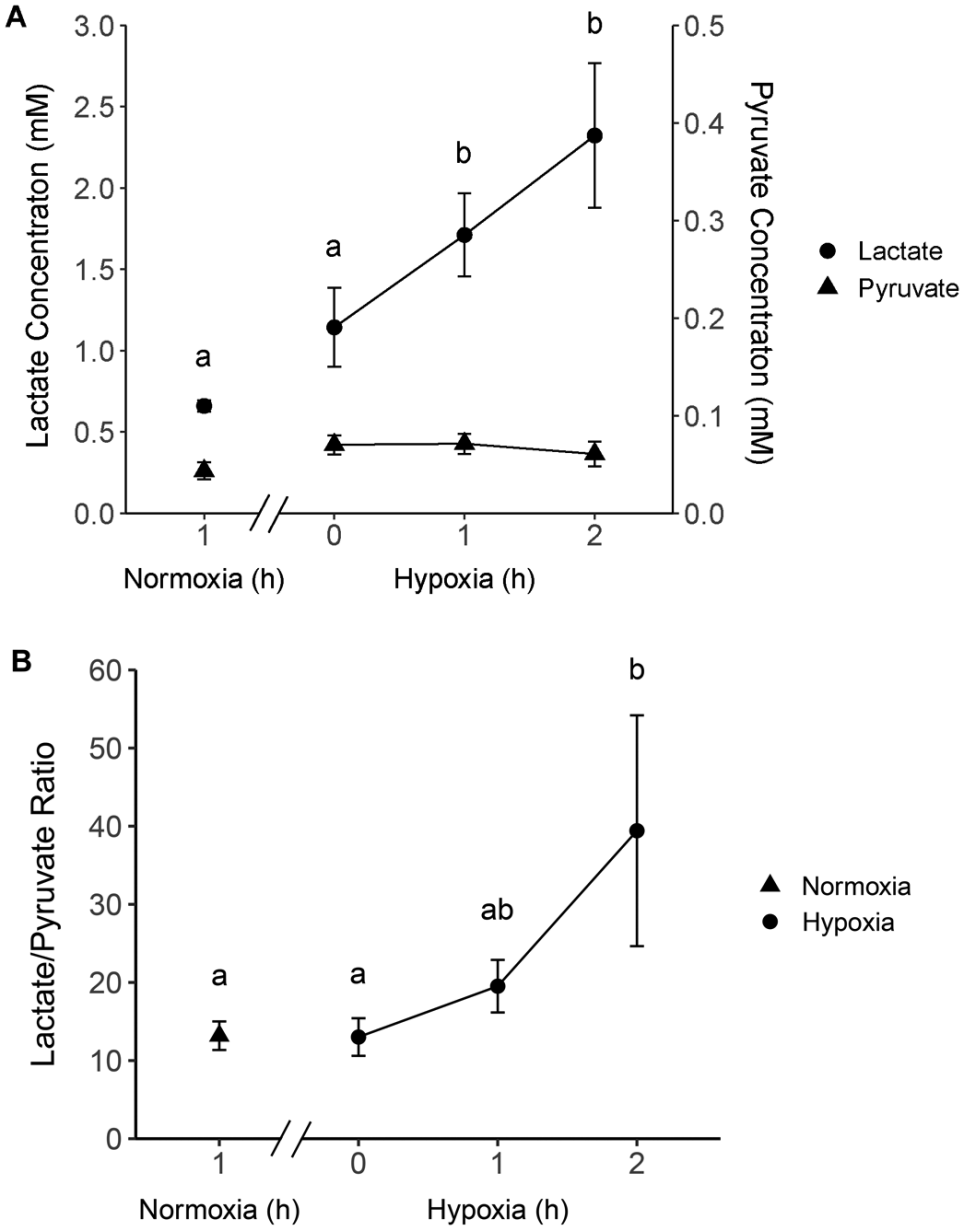
**(A) Lactate and pyruvate levels and (B) Lactate/pyruvate ratio in effluent perfusate sampled at 1 h of normoxic perfusion and 0, 1, and 2 h of hypoxic perfusion.** Data are mean±s.e.m. (*n*=6 except for hypoxia 1 h exposure; *n*=5). Annotations a and b: significant difference (*P*<0.05) in the *post hoc* tests (one-way ANOVA followed by multiple comparisons with Shaffer's adjustment). Double slash (//) on the X axis: another 1 h of normoxia period followed by approximately 30 min of oxygen decrease and acclimation period combined.

### Hypoxia buffer perfusion

The liver was perfused with Tyrode's buffer solution aerated with 95% O_2_ and 5% CO_2_ gas mixture for 2 h to simulate a normoxic condition (pO_2_=88.6±1.0 kPa or O_2_ content=32.1±0.4 mg/L; mean±s.e.m. at 28°C and 0.9% salinity). The oxygen concentration was then decreased by bubbling the buffer with 100% nitrogen gas until it reached a hypoxic level (pO2=∼0.5 kPa or O_2_ content=∼0.2 mg/L). This step was achieved over a duration of ∼10 min followed by a 20 min stabilization period. This was followed by 2 h of perfusion with hypoxic perfusate (Fig. 4B). The pH and temperature of the perfusate were controlled as described for the baseline study. During the normoxia and hypoxia exposure, physiological parameters and perfusate were sampled every 20 min and stored until further analyses.

### Determination of aspartate transferase activity in the perfusate

Aspartate transferase (AST) activity in the effluent perfusate was measured to evaluate hepatic injury. AST activity was quantified colorimetrically based on the manufacturer's protocol for Aspartate Aminotransferase Colorimetric Activity Assay Kit (catalogue number 701640: Cayman Chemical, Ann Arbor, MI, USA). The only deviation from the protocol was that the reaction mixture was incubated at 28°C for 15 min instead of 37°C, as it is in the range of reported optimal temperatures for physiological processes in American alligators and other crocodilians (Cuchens and Clem, 1979; Ertl and Winston, 1998; Seebacher et al., 2003). The absorbance at 340 nm was measured once every minute for 60 min at 28°C using the Cytation 5 imaging reader (US BioTek Laboratories, Shoreline, WA, USA).

### Determination of L-lactate levels in the perfusate

L-lactate is produced from pyruvate by lactate dehydrogenase (LDH) in tissues when oxygen supply is limited hence is a reliable biomarker of anaerobic metabolism (Hochachka and Somero, 2002). The level of L-lactate in perfusate was determined fluorometrically by using the L-Lactate Assay Kit (catalogue number 700510: Cayman Chemical, Ann Arbor, MI, USA). The sample preparation and assay were performed as per the manufacturer's protocol. The fluorescence of reaction mixtures was read using an excitation wavelength of 530–540 nm and an emission wavelength of 585–595 nm using the Cytation 5 imaging reader (US BioTek Laboratories, Shoreline, WA, USA). The L-lactate level was quantified based on the fluorescence strength using the standard curve run at the same time.

### Determination of pyruvate levels in the perfusate

The Pyruvate Assay Kit (catalogue number 700470: Cayman Chemical, Ann Arbor, MI, USA) was used to fluorometrically determine the pyruvate level in the effluent perfusate. The sample preparation and assay were performed as per the manufacturer's protocol. The fluorescence of reaction mixtures was read using an excitation wavelength of 530–540 nm and an emission wavelength of 585–595 nm using the Cytation 5 imaging reader (US BioTek Laboratories, Shoreline, WA, USA). The pyruvate level was quantified based on the fluorescence strength using the standard curve run at the same time. For lactate and pyruvate concentrations, and lactate/pyruvate ratio was calculated for each sampling time point respectively.

### Perfusate biochemistry analysis

The effluent perfusate from one animal used in the hypoxia perfusion study was analyzed for glutamate dehydrogenase (GLDH) activity and glucose level. GLDH was selected as a liver injury biomarker and glucose as a glycolytic metabolism biomarker. The effluent perfusate samples were collected at 0 and 120 min of the normoxia (control) period and at 0 and 120 min of the hypoxia period. The analysis was performed by Texas A&M Veterinary Medical Diagnostic Laboratory using the DxC 700 AU Chemistry Analyzer (Beckman Coulter, Brea, CA, USA).

### Statistical analyses

Data collected during the baseline and hypoxia buffer perfusions were analyzed using R (R Core Team, 2023) and RStudio (Posit team, 2023). Significance was set at *P*<0.05 for all tests unless otherwise stated. The normality of data was checked by performing the Shapiro–Wilk test from the rstatix package (Kassambara, 2023) (α=0.01) whereas homoscedasticity was checked by performing the Breusch–Pegan test or Brown–Forsythe–Levene test. When data failed either assumption, log10 transformation of the dependent variable or a weighted linear mixed effects (lme) model from the nlme package (Pinheiro et al., 2023) was used according to BoxCox test results from the MASS package (Venables and Ripley, 2002). When comparing data between different sampling time points, autocorrelation was checked and addressed for each dependent variable using lme models with AR(1) correlation structure in the nlme package (Pinheiro et al., 2023) based on the results of Akaike's information criterion with correction for small sample size (AICc) (Bartoń, 2023).

For the data from baseline buffer perfusion, each of the measured parameters; AST activity, lactate and pyruvate concentrations, and lactate/pyruvate ratio was compared between sampling time points, respectively. All sampling points were compared by performing repeated one-way ANOVA using a lme model with time as a fixed factor and replicate animals as a random factor. The data of AST activity from hypoxic buffer perfusion was compared between two treatment levels (i.e. normoxia vs hypoxia) and sampling time points, respectively. The significance of treatment, time, and interaction of the two factors was tested by repeated two-way ANOVA using a lme model. When ANOVA indicated the significance of any independent variable, a pairwise *t*-test (one-tailed) was subsequently performed with Benjamini–Hochberg (BH) adjustment. For the analysis of lactate and pyruvate concentrations and lactate/pyruvate ratio in the hypoxic buffer perfusion, different time points were compared by performing repeated one-way ANOVA using a lme model. When ANOVA indicated significance, multiple comparisons with Shaffer's adjustment from the multcomp package (Hothorn et al., 2008) were performed.
